# Complete Genome Sequence of a Suckermouth Catfish Outbreak Isolate, Aeromonas hydrophila Strain LP0103

**DOI:** 10.1128/mra.00408-22

**Published:** 2022-08-22

**Authors:** Kenneth Joseph Bureros, Yu-Che Chiu, Chung-Yi Liou, Cheng-Yu Ma, Liang-Chun Wang

**Affiliations:** a Marine and Pathogenic Microbiology Laboratory, Department of Marine Biotechnology and Resources, College of Marine Science, National Sun Yat-sen University, Kaohsiung City, Taiwan; b Committee of Fisheries Extension Service, College of Marine Science, National Sun Yat-sen University, Kaohsiung City, Taiwan; c Kaohsiung City Animal Protection Office, Kaohsiung City, Taiwan; University of Delaware

## Abstract

Aeromonas hydrophila is the most common opportunistic pathogen that plagues freshwater and euryhaline fishponds. Here, we present the complete genome sequence of A. hydrophila strain LP0103, which was isolated from a bacterial septicemia outbreak among suckermouth catfish (Pterygoplichthys pardalis) at Lotus Pond in Kaohsiung City, Taiwan.

## ANNOUNCEMENT

Aeromonas hydrophila is a Gram-negative, motile, facultatively anaerobic, rod-shaped bacterium linked to motile *Aeromonas* septicemia in various catfish species (1–6). The most cited case was the 2009 A. hydrophila ML09-119 epidemic among farmed channel catfish in Alabama, USA (7). That particular strain was also reported to have an Asian origin (8). In December 2021, over 500 suckermouth catfish per day on average were found dead/moribund at Lotus Pond, Kaohsiung City, Taiwan. We isolated A. hydrophila from the diseased catfish, named this strain LP0103, and sequenced its complete genome.

Wild moribund suckermouth catfish from Lotus Pond were collected, and a portion of each liver was streaked on premade blood agar (CMP0100311; Creative Life Science, Taiwan) and incubated at 27°C for 24 h following the veterinarian standard procedure (9, 10). The isolation showed a single colony type with hemolysis from at least five catfish screened. The Gentra Puregene yeast/bact. kit (Qiagen, Germany) was used to extract DNA from representative colonies isolated from each fish. Sanger sequencing of the 16S rRNA gene using the V1 to V9 region (i.e., with primers 27F [5′-AGAGTTTGATCMTGGCTCAG-3′] and 1492R [5′-TACGGYTACCTTGTTACGACTT-3′]) was performed to confirm that the bacterial colonies were indistinguishable and identified as A. hydrophila.

A single colony of the isolated A. hydrophila was inoculated on brain heart infusion agar and incubated under the aforementioned conditions for subsequent genome sequencing. The same kit was used to extract 5.7 μg genomic DNA from roughly 10^11^ bacteria, and the DNA was subjected it to whole-genome sequencing. A g-TUBE (Covaris, USA) was used to shear 1 μg genomic DNA, which was purified using AMPure PB beads (Pacific Biosciences [PacBio], USA). Following the manufacturer's instructions, the sheared and purified DNA fragments were utilized as a template to prepare a 10-kb high-fidelity sequencing library using the SMRTbell template preparation kit v1.0 (PacBio). After damage and end repairs, the A-tailed inserts were ligated using barcoded overhang adapters, and small insert SMRTbell templates were removed using BluePippin size selection.

Genomics BioSci & Tech Co. (Taipei, Taiwan) performed single-molecule real-time (SMRT) sequencing with 100× coverage on a PacBio Sequel sequencer using a SMRT Cell 1M v3 tray with v3.0 chemistry. The Sequel system was used for the primary filtering analysis, and SMRT Link v9.0 (11) was used for the secondary analysis with default parameters. The pipeline includes consensus sequence determination, assembly, and quality evaluation.

The sequence has 420,087 subreads and an average read length of 4,939 bp. The circular consensus reads were produced using Code Composer Studio v6 (PacBio), and the resulting reads provided base-level resolution with >99.9% accuracy. These reads were then used for assembling and polishing of the genome with hifiasm v0.15.3 (12) and GCpp v2.0.2 (https://github.com/PacificBiosciences/gcpp), respectively. Circlator v1.5.5 (13) was used to correct and circularize the genome, and QUAST v4.6.3 (14) evaluated the assembled genome quality. The A. hydrophila genome comprises 5,023,649 bp in one contig, with a GC content of 60.91%. It contains 4,606 predicted genes, of which 4,398 are protein-coding sequences. A total of 130 tRNAs and 31 rRNA operons were predicted using Prokka v1.12 (15). The final closed-circle version of the A. hydrophila LP0103 genome sequence was submitted to the PGAP v5.3 (16) for annotation, followed by submission to GenBank. Default parameters were used except where otherwise noted.

Further studies of the genome of Aeromonas hydrophila LP0103 and *in vivo* challenge experiments are of interest and may reveal more information on pathogenicity and host specificity for the suckermouth catfish.

### Data availability.

The complete genome sequence of A. hydrophila LP0103 was deposited in GenBank under the accession number CP092906. The raw sequencing reads were deposited in the NCBI Sequence Read Archive (SRA) under accession number SRR18355516.

## References

[1] El-Son MAM, Nofal MI, Abdel-Latif HMR. 2021. Co-infection of *Aeromonas hydrophila* and *Vibrio parahaemolyticus* isolated from diseased farmed striped mullet (*Mugil cephalus*) in Manzala, Egypt: a case report. Aquaculture 530:735738. doi:10.1016/j.aquaculture.2020.735738.

[2] Zhang XT, Zhang GR, Shi ZC, Yuan YJ, Zheng H, Lin L, Wei KJ, Ji W. 2017. Expression analysis of nine Toll-like receptors in yellow catfish (*Pelteobagrus fulvidraco*) responding to *Aeromonas hydrophila* challenge. Fish Shellfish Immunol 63:384–393. doi:10.1016/j.fsi.2017.02.021. 28223111

[3] Baumgartner WA, Ford L, Hanson L. 2017. Lesions caused by virulent *Aeromonas hydrophila* in farmed catfish (*Ictalurus punctatus* and *I. punctatus* × *I. furcatus*) in Mississippi. J Vet Diagn Invest 29:747–751. doi:10.1177/1040638717708584. 28482758

[4] Abdelhamed H, Ibrahim I, Baumgartner W, Lawrence ML, Karsi A. 2017. Characterization of histopathological and ultrastructural changes in channel catfish experimentally infected with virulent *Aeromonas hydrophila*. Front Microbiol 8:1519. doi:10.3389/fmicb.2017.01519. 28861049 PMC5559642

[5] Le TS, Nguyen TH, Vo HP, Doan VC, Nguyen HL, Tran MT, Tran TT, Southgate PC, Kurtböke Dİ. 2018. Protective effects of bacteriophages against *Aeromonas hydrophila* causing motile *Aeromonas* septicemia (MAS) in striped catfish. Antibiotics 7:16. doi:10.3390/antibiotics7010016. PMC5872127 29495337

[6] Bandeira G, Jr, de Freitas Souza C, Descovi SN, Antoniazzi A, Cargnelutti JF, Baldisserotto B. 2019. *Aeromonas hydrophila* infection in silver catfish causes hyperlocomotion related to stress. Microb Pathog 132:261–265. doi:10.1016/j.micpath.2019.05.017. 31078710

[7] Tekedar HC, Waldbieser GC, Karsi A, Liles MR, Griffin MJ, Vamenta S, Sonstegard T, Hossain M, Schroeder SG, Khoo L, Lawrence ML. 2013. Complete genome sequence of a channel catfish epidemic isolate, *Aeromonas hydrophila* strain ML09-119. Genome Announc 1:e00755-13. doi:10.1128/genomeA.00755-13. 24051325 PMC3778208

[8] Hossain MJ, Sun D, McGarey DJ, Wrenn S, Alexander LM, Martino ME, Xing Y, Terhune JS, Liles MR. 2014. An Asian origin of virulent *Aeromonas hydrophila* responsible for disease epidemics in United States-farmed catfish. mBio 5:e00848-14. doi:10.1128/mBio.00848-14. 24895303 PMC4049099

[9] Johnson M. 1993. The veterinary approach to channel catfish, p 249–270. *In* Brown L (ed), Aquaculture for veterinarians: fish husbandry and medicine, 1st ed. Pergamon Press, Oxford, United Kingdom.

[10] Topić Popović N, Teskeredžić E, Strunjak-Perović I, Čož-Rakovac R. 2000. *Aeromonas hydrophila* isolated from wild freshwater fish in Croatia. Vet Res Commun 24:371–377. doi:10.1023/A:1006418116155. 11014606

[11] Pacific Biosciences. 2020. Smart Link use guide: Sequel system. Pacific Biosciences, Menlo Park, CA. https://www.pacb.com/wp-content/uploads/SMRT_Link_User_Guide_v90.pdf.

[12] Cheng H, Concepcion GT, Feng X, Zhang H, Li H. 2021. Haplotype-resolved de novo assembly using phased assembly graphs with hifiasm. Nat Methods 18:170–175. doi:10.1038/s41592-020-01056-5. 33526886 PMC7961889

[13] Hunt M, De Silva N, Otto TD, Parkhill J, Keane JA, Harris SR. 2015. Circlator: automated circularization of genome assemblies using long sequencing reads. Genome Biol 16:294. doi:10.1186/s13059-015-0849-0. 26714481 PMC4699355

[14] Gurevich A, Saveliev V, Vyahhi N, Tesler G. 2013. QUAST: quality assessment tool for genome assemblies. Bioinformatics 29:1072–1075. doi:10.1093/bioinformatics/btt086. 23422339 PMC3624806

[15] Seemann T. 2014. Prokka: rapid prokaryotic genome annotation. Bioinformatics 30:2068–2069. doi:10.1093/bioinformatics/btu153. 24642063

[16] Tatusova T, DiCuccio M, Badretdin A, Chetvernin V, Nawrocki EP, Zaslavsky L, Lomsadze A, Pruitt KD, Borodovsky M, Ostell J. 2016. NCBI Prokaryotic Genome Annotation Pipeline. Nucleic Acids Res 44:6614–6624. doi:10.1093/nar/gkw569. 27342282 PMC5001611

